# Challenges in Gene Therapy for Somatic Reverted Mosaicism in X-Linked Combined Immunodeficiency by CRISPR/Cas9 and Prime Editing

**DOI:** 10.3390/genes13122348

**Published:** 2022-12-13

**Authors:** Yujuan Hou, Guillermo Ureña-Bailén, Tahereh Mohammadian Gol, Paul Gerhard Gratz, Hans Peter Gratz, Alicia Roig-Merino, Justin S. Antony, Andrés Lamsfus-Calle, Alberto Daniel-Moreno, Rupert Handgretinger, Markus Mezger

**Affiliations:** 1 Department of Pediatrics I, Hematology and Oncology, University Children’s Hospital, University of Tübingen, 72076 Tuebingen, Germany; 2 MaxCyte Inc., Rockville, MD 20850, USA

**Keywords:** *IL2RG*, SCID, CRISPR/Cas9, ssODN, prime editing, somatic mosaicism

## Abstract

X-linked severe combined immunodeficiency (X-SCID) is a primary immunodeficiency that is caused by mutations in the interleukin-2 receptor gamma *(IL2RG)* gene. Some patients present atypical X-SCID with mild clinical symptoms due to somatic revertant mosaicism. CRISPR/Cas9 and prime editing are two advanced genome editing tools that paved the way for treating immune deficiency diseases. Prime editing overcomes the limitations of the CRISPR/Cas9 system, as it does not need to induce double-strand breaks (DSBs) or exogenous donor DNA templates to modify the genome. Here, we applied CRISPR/Cas9 with single-stranded oligodeoxynucleotides (ssODNs) and prime editing methods to generate an in vitro model of the disease in K–562 cells and healthy donors’ T cells for the c. 458T>C point mutation in the *IL2RG* gene, which also resulted in a useful way to optimize the gene correction approach for subsequent experiments in patients’ cells. Both methods proved to be successful and were able to induce the mutation of up to 31% of treated K–562 cells and 26% of treated T cells. We also applied similar strategies to correct the *IL2RG* c. 458T>C mutation in patient T cells that carry the mutation with revertant somatic mosaicism. However, both methods failed to increase the frequency of the wild-type sequence in the mosaic T cells of patients due to limited in vitro proliferation of mutant cells and the presence of somatic reversion. To the best of our knowledge, this is the first attempt to treat mosaic cells from atypical X-SCID patients employing CRISPR/Cas9 and prime editing. We showed that prime editing can be applied to the formation of specific-point *IL2RG* mutations without inducing nonspecific on-target modifications. We hypothesize that the feasibility of the nucleotide substitution of the *IL2RG* gene using gene therapy, especially prime editing, could provide an alternative strategy to treat X-SCID patients without revertant mutations, and further technological improvements need to be developed to correct somatic mosaicism mutations.

## 1. Introduction

X-linked severe combined immunodeficiency (X-SCID) is an X-chromosome recessive primary immunodeficiency disorder caused by mutations in the interleukin-2 receptor gamma gene (*IL2RG*). The *IL2RG* gene encodes the interleukin common gamma chain (γc) of different cytokine receptors, including interleukin (IL)-2, IL-4, IL-7, IL-9, IL-15, and IL-21. The γc plays a critical role in lymphocyte development and immune function [1]. Mutations in the *IL2RG* gene cause an impairment of the immune system due to the severe scarcity of T cells and NK cells in patients with the typical form of the disease [2]. Without any treatment, the risk of death is very high within the first year of life [2,3]. On the other hand, atypical X-SCID patients present a milder form of immunodeficiency due to either hypomorphic mutations [4] or natural correction induced by somatic reversions [5,6,7,8,9,10,11,12,13]. The current treatment of X-SCID includes prophylaxis for infections to ensure the safety of patients and hematopoietic stem cell transplantation (HSCT) to reconstruct their immune function [2]. Allogeneic HSCT (allo-HSCT) has remarkably improved the survival rate for X-SCID patients; however, it has several disadvantages, such as the difficulty of finding HLA-matched sibling donors and the risk of graft rejection [14,15]. Subsequently, viral, vector-based gene therapy for autologous HSCT (auto-HSCT) has emerged. Although considerable clinical benefits have been reported in treated patients, the risk of insertional mutagenesis is considered a major downside of this method [16,17,18].

CRISPR/Cas9 is a modern multifunctional gene editing tool that can provide treatment alternatives for genetic diseases such as X-SCID [19,20]. Specific gene replacement and correction by CRISPR/Cas9 systems demand a high rate of homology-directed repair (HDR) of double-strand breaks (DSBs), but this is a rare cellular event due to the dominant role of the non-homologous end joining repair pathway (NHEJ) during the cell cycle [21]. State-of-the-art prime editing technology can bypass this limitation, as it does not require DSBs induction nor the presence of HDR donor templates to modify the genome. Prime editing is a more accurate genome editing technique that directly completes the genomic edit at the specified DNA site using a catalytically impaired Cas9 fused to an engineered reverse transcriptase (RT), programmed with a prime editing guide RNA (pegRNA) specifying the target site and encoding the desired modification [22,23]. Compared with the CRISPR/Cas9 system, prime editing offers higher efficiency and fewer off-target byproducts [22].

The fact that the survival rate in atypical X-SCID patients with spontaneous revertant mutations is higher than in typical X-SCID cases shows that even the partial restoration of immune system functions by gene therapy can have positive therapeutic effects [24]. Recently, we described the clinical and molecular characteristics of a novel *IL2RG* single-nucleotide variant, c.458T>C; p.Ile153Thr, in three brothers (P1, P2, P3) with mild symptoms of immunodeficiency. We could detect mosaic T cells with revertant mutations (the *IL2RG* c.458T>C mutation reverts to the wild-type) in these patients [13]. In this study, we employed two genome editing technologies, CRISPR/Cas9 and prime editing, to target the *IL2RG* c.458T>C mutation in the mosaic T cells of atypical X-SCID patients.

## 2. Materials and Methods

### 2.1. Ethics Statement

Human CD3^+^ T cells from peripheral blood were obtained following informed written consent under the Declaration of Helsinki and the approval of the Institutional Review Board of the University of Tübingen Ethics Committee (No. 928/2020BO2).

### 2.2. The Components for Gene-Editing

CRISPR/Cas9 single-guide RNAs (sgRNAs) for targeting *IL2RG* were designed by CHOPCHOP [25] online software (https://chopchop.cbu.uib.no/) (accessed on 31 January 2020), and the sgRNA of *TRAC* was gained from the literature [26]. All sgRNAs were synthesized by Integrated DNA Technologies (IDT, Coralville, IA, USA) (Table S1). Single-stranded oligonucleotides (ssODNs) donors were designed with the Horizon HDR donor designer tool (https://horizondiscovery.com/en/ordering-and-calculation-tools/ediTr-hdr-donor-designer-oligo) (accessed on 31 November 2020) and synthesized by Metabion (Planegg/Steinkirchen, Germany) (Table S2). Prime editing guide RNAs (pegRNAs) were designed with the pegFinder [27] online tool (http://pegfinder.sidichenlab.org/) (accessed on 25 February 2021) and synthesized by IDT (Coralville, IA, USA) (Table S3).

pCMV-PE2 (PE2) and pCMV-PE2-P2A-GFP (PE2-GFP) plasmids were purchased from Addgene (Watertown, MA, USA) [22]. Both plasmids were purified with the Plasmid Maxiprep Kit (QIAGEN, Dusseldorf, Germany) following the instruction manual. The plasmids were linearized by restriction endonuclease *PmeI* (New England BioLabs, NEB, Ipswich, MA, USA) at 37 °C for 2 h. In vitro mRNA transcription was performed using the MEGAscript T7 transcription kit (Thermo Fisher Scientific, Waltham, MA, USA), Ambion^®^ Anti-Reverse Cap Analog (ARCA, 3′-O-methyl-m75′Gppp5′G) (Thermo Fisher Scientific, Waltham, MA, USA), and Poly (A) Tailing Kit (Thermo Fisher Scientific, Waltham, MA, USA) following the instructions of the manufacturers. The purification of mRNA was performed using LiCl precipitation. Subsequently, the concentrations of mRNA were measured with NanoDrop (Thermo Fisher Scientific, Waltham, MA, USA), and the quality of produced mRNA was checked in 1% UltraPure™ Agarose (ThermoFisher Scientific, Waltham, MA, USA) gel through electrophoresis. The DsRed mRNA was synthesized in vitro according to a previously described protocol [28]. The produced mRNAs were stored at −80 °C.

### 2.3. Cell Culture

CD3^+^ T cells were isolated from the heparinized peripheral blood of patients and healthy donors following our previously published methods [13]. The purity of the T cell population was >90% for all isolations. T cells were cultured in TexMACS medium (Miltenyi Biotec, Bergisch Gladbach, Germany) supplemented with 10% Fetal Bovine Serum (FBS, Biochrom, Berlin, Germany), 1% Penicillin/Streptomycin (P/S, Thermo Fisher Scientific, Waltham, MA, USA), and different cytokines (10 ng/mL IL-7 and 5 µg/mL IL-15 for healthy donors’ cells; 50 units/mL IL-2 for patients’ cells; Miltenyi Biotec, Bergisch Gladbach, Germany). In total, 10 µL of TransAct (Miltenyi Biotec, Bergisch Gladbach, Germany) per 10^6^ cells was added after isolation to stimulate the expansion of T cells in vitro [13].

The K–562 cell line (Sigma-Aldrich, Burlington, MA, USA) was cultured in RPMI-1640 medium (Sigma-Aldrich, Burlington, MA, USA) supplemented with 10% FBS (Biochrom, Berlin, Germany), 1% L-glutamine (Biochrom, Berlin, Germany), and 1% P/S (Thermo Fisher Scientific, Waltham, MA USA).

All cells were cultured in the incubator at 37 °C with 5% CO_2_.

### 2.4. Genomic Analysis

Genomic DNA (gDNA) was isolated using the NucleoSpin Tissue kit (Macherey Nagel, Düren, Germany) following the manufacturer’s instructions. After measuring the concentration, 5 ng/µL gDNA, 250 nM primers, and GoTaq^®^ Green Master Mix (Promega, Madison, WI, USA) were used to amplify the genomic region (409 bp) covering the *IL2RG* c.458T>C site (Table S4). The PCR protocol consisted of 95 °C for 2 min followed by 40 cycles of 95 °C for 40 s, 62 °C for 30 s, and 68 °C for 1 min. A QIAquick PCR purification kit (QIAGEN, Dusseldorf, Germany) was used for PCR product purification. The resulting product was mixed with forward primer and sent to Eurofins Genomics (Ebersberg, Germany) for Sanger sequencing.

### 2.5. In Vitro CRISPR/Cas9 Cutting Assay

As previously described [29], the reaction, including a PCR product (100 nM) (PCR protocol described in Section 2.4) and a ribonucleoprotein (RNP) complex consisting of sgRNAs (200 nM) and Cas9 nuclease (100 nM) (IDT, Coralville, IA, USA), was incubated at 37 °C for 2 h. The reaction was stopped by adding proteinase K (Macherey Nagel, Düren, Germany) for 10 min at 56 °C. Then, 1% agarose (Lonza, Basel, Switzerland) gel electrophoresis was used to visualize the resulting products.

### 2.6. Screening of sgRNAs

Electroporation was performed in the K–562 cells and CD3^+^ T cells of the healthy donors (3 days after isolation and TransAct activation) and patients (1 day after isolation and TransAct activation). The RNP was mixed with sgRNAs (9 µM) of *IL2RG* or *TRAC* and Cas9 nuclease (4.5 µM) (IDT, Coralville, IA, USA), and incubated at room temperature for 15 min before transfection. In total, 100,000 cells were transfected using a Neon Electroporation System (Thermo Fisher Scientific, Waltham, MA USA) and a 10 µL Neon transfection kit (Thermo Fisher Scientific, Waltham, MA USA) with the following settings: 1450 V, 10 ms, 3 pulses (K–562 cells); 1800 V, 10 ms, 3 pulses (T cells). On day 2 (patient T cells) or day 5 (K–562 and healthy donors’ T cells) of post-transfection, the gDNA was isolated for Sanger sequencing.

### 2.7. Cell Proliferation Assay

A cell proliferation assay was carried out in the transfected T cells of healthy donors. The cell number was measured with FACS Calibur (BD Biosciences, Franklin Lakes, NJ, USA) and analyzed with FlowJo software (BD Biosciences, Franklin Lakes, NJ, USA). Cell proliferation rate is presented as the fold-change between the cell number on dayZ and day1.

### 2.8. Transfection of CRISPR/Cas9-ssODN

Before electroporation, T cells were activated and expanded three days post-isolation for healthy donors and one day for patients’ cells. For electroporation, RNP complexes (sgRNAs and Cas9) in a molar ratio of 2:1 (3 µM:1.5 µM in K–562 cells; 4 µM:2 µM in T cells) were incubated at room temperature for 15 min, and then, ssODNs were added to the complex at 6 µM. Subsequently, 2.5 × 10^6^ K–562 cells and 1.5 × 10^6^ T cells were electroporated with ExPERT GTx^®^ using R-50 × 3 processing assemblies with the “K562” and “Expanded T cell 3” programs (MaxCyte^®^, Rockville, MD, USA). Subsequently, the K–562 cells were seeded in prewarmed 6-well plates to recover for 30 min in the incubator. Then, fresh RPMI-1640 medium without supplements was added to the cultured cells (1 × 10^6^ cells/mL). Transfected T cells were seeded in prewarmed 24-well plates to recover for 30 min in the incubator and cultured with fresh TexMACS medium without antibiotics and interleukins (2 × 10^6^ cells/mL). After 4 h, TexMACS medium, with 2X IL-7 (20 ng/mL) and IL-15 (10 ng/mL) for healthy donors’ T cells or IL-2 (100 units/mL) for patients T cells, was added to reach the final concentration of 1 × 10^6^ cells/mL. On day 5 post-transfection, the gDNA was isolated for the subsequent PCR and Sanger sequencing of the target sequence.

### 2.9. Transfection of Prime Editing (PE2)

In total, 1.5 × 10^6^ activated and expanded T cells (three days post-isolation for healthy donors’ cells; one day post-isolation for patients’ cells) were transfected with 5 µg of mRNA (PE2 or PE2-GFP) and pegRNA (3 µM) using the “Expanded T cell 3” program of the GTx^TM^ Transfection System and R-50×3 processing assemblies (MaxCyte, Rockville, MD, USA). After electroporation, cells were treated with corresponding mediums to recover and proliferate as previously stated (Section 2.9). PE2 or PE2-GFP mRNA (1 µg) and pegRNA (900 nM) were utilized to transfect 3 × 10^5^ K–562 cells using the Neon Electroporation System (Thermo Fisher Scientific, Waltham, MA USA) and a 10 µL Neon transfection kit (Thermo Fisher Scientific, Waltham, MA USA) using the following electroporation settings: 1450 V, 10 ms, 3 pulses. On day 2 after transfection, gDNA was isolated from K–562 and T cells to analyze the editing efficiency.

### 2.10. Editing Efficiency Analysis

#### 2.10.1. DsRed Expression

DsRed mRNA was used as a marker of transfection efficiency in all experiments. For electroporation, 100 µg/mL of mRNA was transfected to cells using ExPERT GTx^®^ (MaxCyte^®^, Rockville, MD, USA) or the Neon Electroporation System (Thermo Fisher Scientific, Waltham, MA USA). The mRNA expression was detected by FACS Calibur (BD Biosciences, Franklin Lakes, NJ, USA) 24 h post-transfection and analyzed by FlowJo software (BD Biosciences, Franklin Lakes, NJ, USA).

#### 2.10.2. GFP Expression

Twenty-four hours after electroporation, 100 µL of cells transfected with PE2-GFP mRNA and pegRNA was collected to check for GFP expression using FACS Calibur (BD Biosciences, Franklin Lakes, NJ, USA) and analyzed by FlowJo software (BD Biosciences, Franklin Lakes, NJ, USA).

#### 2.10.3. Sanger Sequencing

The gDNA analysis was carried out with Sanger sequencing (Section 2.4). The ICE analysis for the frequencies of the indel, HDR, and induced mutation based on the Sanger sequencing was performed using the ICE SYNTHEGO online tool [30] (https://ice.synthego.com) (accessed on 29 April 2020 to 1 October 2022).

#### 2.10.4. Restriction Fragment Length Polymorphism (RFLP) Analysis

RFLP was performed to detect the induced mutation on K–562 and healthy donors’ T cells. In total, 200 ng of PCR product was digested with 5 units of *DpnII* (NEB, Ipswich, MA, USA) and incubated at 37 °C for 2 h. Then, 5 µL of digested and undigested samples was mixed with 1 µL DNA Gel Loading Dye (6X) (NEB, Ipswich, MA, USA) and was loaded on 2.5% agarose gel for electrophoresis. The wild-type sequence was expected to generate four fragments (167 bp, 143 bp, 55 bp, and 44 bp), whereas the mutated one was expected to generate three bands (222 bp, 143 bp, and 44 bp).

#### 2.10.5. Droplet Digital PCR (ddPCR) Analysis

A master mix with a 20 µL volume was prepared by adding DNA (50 ng), primers (900 nM) (Table S5), probes (250 nM) (Table S5), 2X ddPCR Supermix for Probes (no dUTP) (Bio-Rad Laboratories, Hercules, CA, USA), and 5 units of restriction endonuclease *MseI* (NEB, Ipswich, MA, USA); then, it was incubated at room temperature for 15 min. In total, 70 µL of droplet-generating oil (Bio-Rad Laboratories, Hercules, CA, USA) was added to the sample and subsequently loaded to a DG8 (Bio-Rad Laboratories, Hercules, CA, USA) to separate DNA into droplets using a QX200 ddPCR droplet generator (Bio-Rad Laboratories, Hercules, CA, USA). Next, 42 µL of droplet mixture was transferred into a 96-deep-well reaction plate and was sealed with PX1 PCR Plate Sealer (Bio-Rad Laboratories, Hercules, CA, USA). Finally, the amplification was performed by a thermocycler (Bio-Rad Laboratories, Hercules, CA, USA) with the following program: 95 °C for 10 min, 40 cycles of 94 °C for 30 s and 55 °C for 1 min, and 98 °C for 10 min. The PCR products were detected by the QX200 Droplet Reader (Bio-Rad Laboratories, Hercules, CA, USA) and analyzed using the QuantaSoft software, version 1.7.4 (Bio-Rad Laboratories, Hercules, CA, USA). The difference in the ratio of *IL2RG* (copies/µL)/*RPP30* (copies/µL) between the edited samples and controls was calculated as the correction efficiency.

### 2.11. Statistical Analysis

Graph Pad Prism 9.1.2 software (GraphPad Software, San Diego, CA, USA) was used for statistical analysis. An ordinary one-way ANOVA test was applied to compare the proliferation of the healthy donors’ T cells between post-transfection and non-transfection (****, *p* < 0.0001).

## 3. Results

### 3.1. Reduced Frequency of Mutant c.458T>C IL2RG in Mosaic T Cells after In Vitro Cultivation

**Figure 1:**
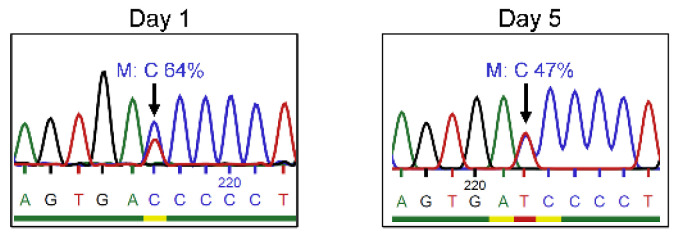
Frequency of the *IL2RG* c.458T>C mutation in mosaic T cells. Expanded CD3^+^ T cells of P1 were harvested to purify gDNA on days 1 and 5 post-isolation, separately. Sanger sequencing showed the frequency of the mutant (M) base, C, was 64% on day 1 and 47% on day 5 in the mosaic T cells (the blue, red, green, and black peaks stand in place of bases C, T, A, and G respectively).

Following the observations reported in our previous publication regarding the presence of revertant mutations in the T cells of three brothers [13], genetic analyses were performed to study mutation frequency in the expanded CD3^+^ T cells of P1. The frequency of the mutant sequence was reduced after an in vitro culture (64% on day 1 vs. 47% on day 5) (Figure 1).

### 3.2. Gene Correction by CRISPR/Cas9-ssODN Approach

#### 3.2.1. sgRNAs Screening

**Figure 2:**
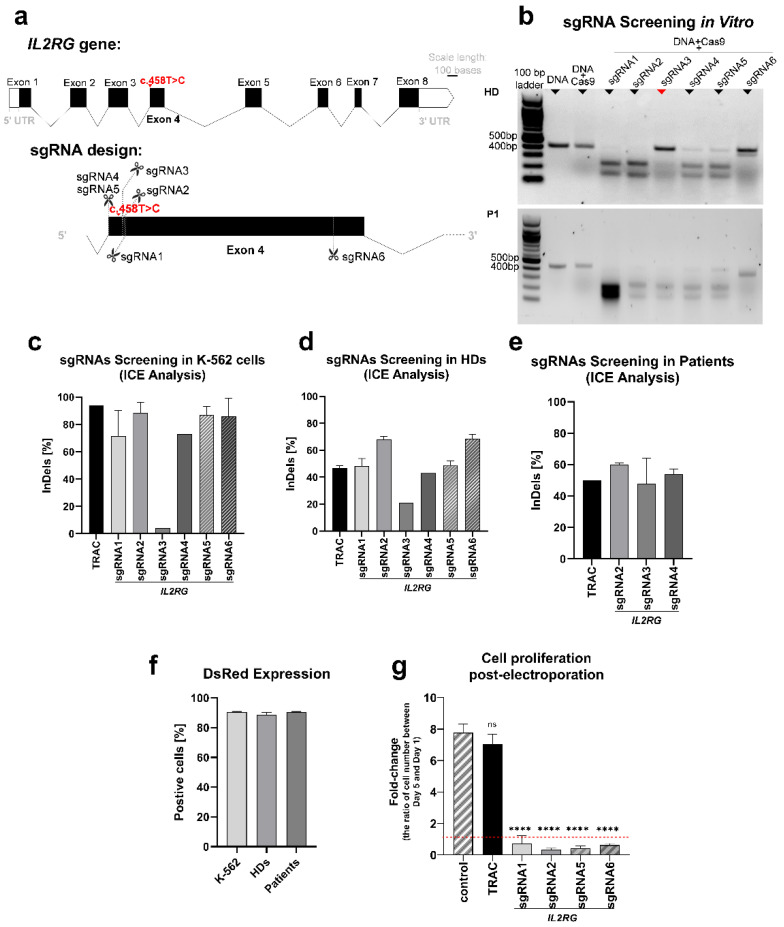
Design and screening of sgRNAs. (**a**) Schematic representations of the *IL2RG* gene and designed sgRNAs were created in the Exon-Intron Graphic Maker (http://wormweb.org/exonintron) and modified in PowerPoint. the *IL2RG* gene’s eight exons are depicted: the c.458T>C mutation is presented on Exon4. The cutting sites of all designed sgRNAs are highlighted. sgRNA1 and 5 target the wild-type sequence; sgRNA3 and 4 target the mutant sequence; and sgRNA2 and 6 target both sequences. (**b**) In vitro CRISPR/Cas9 cutting assay. The visualization of the agarose gel showed that the PCR products (409 bp) were cut into fragments of different sizes after RNP incubation for 2 h (bands with 246 bp and 163 bp for sgRNA1; 251 bp and 158 bp for sgRNA2; 250 bp and 159 bp for sgRNA3; 243 bp and 166 bp for sgRNA4; 243 bp and 166 bp for sgRNA5; 364 bp and 45 bp for sgRNA6). sgRNA1, 2, 4, 5, and 6 similarly cut the target DNA amplicon of both the healthy donor (HD) and P1, while sgRNA3 only worked for the DNA of P1. (**c**) sgRNA screening of K–562 cells (n = 2). All sgRNAs led to a more than 70% indel frequency, except sgRNA3 (4%). (**d**) sgRNA screening of T cells in healthy donors (n = 3). sgRNA3 generated a 21% indel rate, whereas the rest of the sgRNAs generated a more than 60% indel rate. (**e**) sgRNA screening of T cells in patients (n = 2, P1 and P2). sgRNAs induced a 32–64% indel formation. (**f**) DsRed protein expression was detected 24 h post-transfection with flow cytometry as 90% in K–562 and T cells. (**g**) The proliferation rate of transfected T cells (healthy donors, n = 3) is calculated according to the fold-change (ratio) of the cell number between day 5 and day 1 post-electroporation. Compared with non-transfected cells, the proliferation rate was reduced significantly in cells transfected with *IL2RG* sgRNAs and Cas9 (****, *p* < 0.0001; ordinary one-way ANOVA test) but not in cells transfected with *TRAC* sgRNA and Cas9 (ns, *p* > 0.05; ordinary one-way ANOVA test). Mean ± SEM of biologically independent experiments is shown.

Different sgRNAs were designed to target Exon4 in *IL2RG* to target the c.458T>C; p.Ile153Thr mutation (Figure 2a). Due to the existence of somatic mosaicism and revertant mutation, we designed sgRNAs to target both wild-type and mutant sequences. SgRNA1 and sgRNA5 were designed to target the wild-type sequence. SgRNA2 and sgRNA6 target both the wild-type and mutant sequence, while sgRNA3 and sgRNA4 target the mutant in a sequence-specific manner. The sgRNA3 was designed so the PAM containing the mutant base to avoid cutting the wild-type sequence or recutting the corrected one.

In order to select the most suitable sgRNA candidates for further experiments, we first validated the cutting potential of the synthesized sgRNAs. For this aim, all six sgRNAs were screened in a cell-free in vitro cutting assay. Only the cutting efficiency of sgRNA3 showed a difference between the healthy and patient DNA, as it did not cut the wild-type sequence (Figure 2b). Then, screening the sgRNAs in the K–562 cells showed high frequencies for the indel analysis in all sgRNAs (more than 70%), except sgRNA3 (4%) (Figure 2c). Moreover, screening the sgRNAs in the T cells of healthy donors showed 21% indel frequency for gRNA3 and up to 72% for the other sgRNAs (Figure 2d). In the following steps, sgRNA2 (with high efficiency in K–562 cells and healthy donors’ T cells), sgRNA3, and sgRNA4 (targeting the mutant sequence) were selected to transfect the T cells of patients. The indel formation was 32–64% in the T cells of patients transfected with sgRNA2, 3, and 4 (Figure 2e). In all experiments, a TRAC RNP electroporated sample was employed as a positive transfection control (Figure 2c–e), and a DsRed mRNA electroporated sample was used as a reporter of transfection efficiency (Figure 2f). Furthermore, cell proliferation was monitored in the transfected T cells of healthy donors. The proliferation of cells transfected with RNP complex (*IL2RG* sgRNAs and Cas9) at day 5 post-electroporation significantly reduced in comparison with the non-transfected cells (*p* < 0.0001), but there was no discernible decrease in cells transfected with TRAC sgRNA and Cas9 (Figure 2g).

#### 3.2.2. Inducing *IL2RG* Mutation in K–562 and T cells

**Figure 3:**
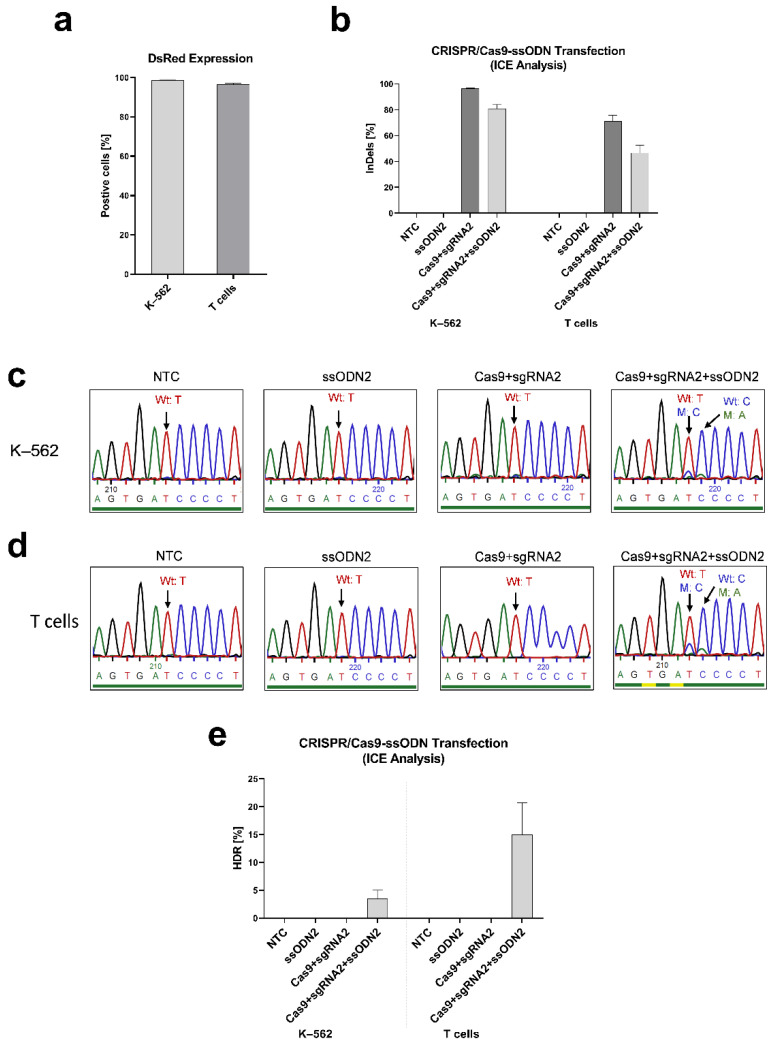
CRISPR/Cas9-ssODN transfection to induce mutation in K–562 cells and T cells from healthy donors. The transfection of RNP (*IL2RG* sgRNA2 and Cas9)-ssODN2 to induce *IL2RG* mutation in the K–562 cells (n = 2) and T cells of healthy donors (n = 3) was performed with MaxCyte^®^. (**a**) DsRed was expressed in more than 96% of K–562 cell and T cell control samples. (**b**) The indel frequencies were 96–97% (RNP transfection) and 78–84% (RNP-ssODN transfection) in K–562 cells and 66–80% (RNP transfection) and 35–54% (RNP-ssODN transfection) in T cells. Sanger sequencing results of (**c**) K–562 cells and (**d**) T cells transfected with RNP-ssODN showed induced mutant nucleotides of c.458C (targeting inducing mutant base) and c.459A (silent blocking mutation) (the blue, red, green, and black peaks stand in place of bases C, T, A, and G respectively). (**e**) HDR efficiency quantified by ICE analysis based on Sanger sequencing showed 3.5 ± 1.5% HDR in K–562 cells and 15.0 ± 5.7% HDR in T cells. Mean ± SEM of biologically independent experiments is shown.

To evaluate whether our designed CRISPR/Cas9 and ssODN system is able to modify the *IL2RG* gene in the c.458 position, we first induced the *IL2RG* c.458T>C mutation in K–562 cells and the T cells of healthy donors. We designed two ssODNs carrying the mutant base. ssODN1 includes the mutant nucleotide (c.458C), and ssODN2 carries this mutant nucleotide and a silent CRISPR/Cas9-blocking mutation (c.459A) to avoid recutting and improve the editing efficiency [31,32]. DsRed control samples expressed DsRed in more than 96% of K–562 cells and T cells (Figure 3a). In RNP-ssODN-transfected samples, the indel frequencies were 78–84% in K–562 cells and 35–54% in T cells (Figure 3b). The mutant nucleotides (c.458T>C and c.459C>A) were detected using Sanger sequencing on transfected K–562 cells (Figure 3c) and T cells (Figure 3d). The HDR efficiency was quantified using ICE analysis as 3.5 ± 1.5% in K–562 cells and 15.0 ± 5.7% in T cells (Figure 3e).

#### 3.2.3. Correcting the IL2RG c.458T>C Mutation in Mosaic T Cells

**Figure 4:**
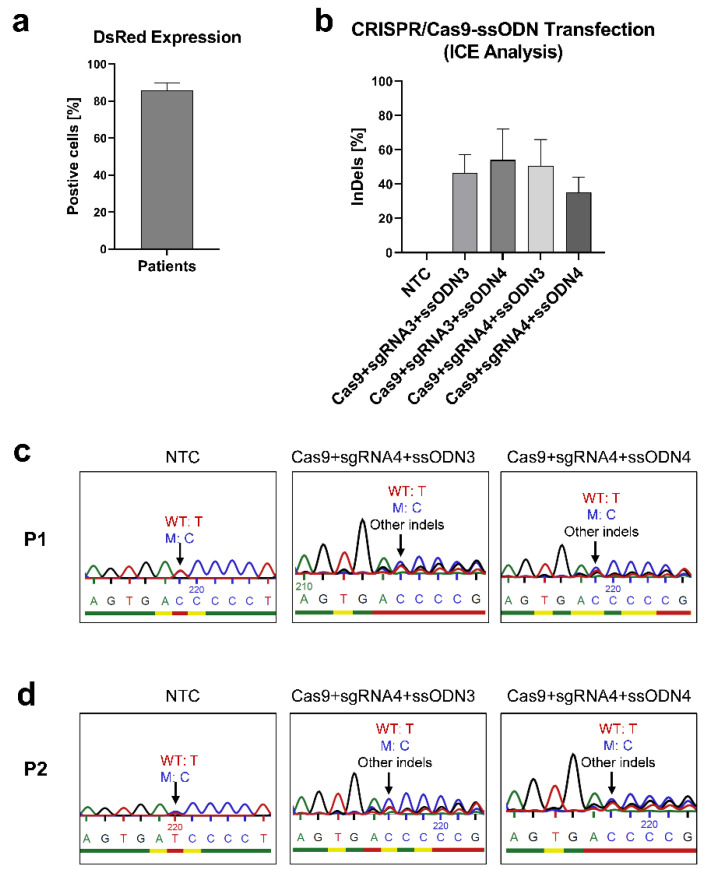
CRISPR/Cas9-ssODN transfection in mosaic T cells to correct the *IL2RG* c.458T>C mutation. T cells of patients (n = 2, P1 and P2) were transfected with RNP (*IL2RG* sgRNA3/4 and Cas9)-ssODN3/4 to correct the c.458T>C *IL2RG* mutation with MaxCyte^®^. (**a**) DsRed mRNA was expressed in up to 90% of cells. (**b**) The indel frequency of edited samples with different RNPs and ssODNs was 26–72%. Mean ± SEM of biologically independent experiments is shown. The Sanger sequencing of non-transfected and transfected T cells with the Cas9, sgRNA4, and ssODN3/4 T cells of (**c**) P1 and (**d**) P2. (The blue, red, green, and black peaks stand in place of bases C, T, A, and G respectively). Compared with the unedited cells, the indel generation was visible, but no higher frequency of the wild-type nucleotide was observed in the edited samples.

To correct the *IL2RG* c.458T>C mutation in mosaic T cells, two ssODNs carrying the wild-type nucleotide (c.458T) were designed. The RNP complex (Cas9 with sgRNA3 or sgRNA4) and ssODN (ssODN3 or ssODN4) were transfected into patient T cells. Up to 90% of control cells expressed DsRed (Figure 4a), and the transfected cells with RNP-ssODN showed 26–72% indel frequencies (Figure 4b). However, no higher frequency of the wild-type nucleotide in the transfected T cells with RNP-ssODN could be detected in comparison with the unedited T cells (Figure 4c,d). This was further confirmed by the fact that neither ICE analysis nor ddPCR analysis could detect an HDR frequency in the transfected T cells from patients.

### 3.3. Gene Editing by Prime Editing

#### 3.3.1. Inducing the *IL2RG* c.458T>C Mutation in K–562 and T Cells

**Figure 5:**
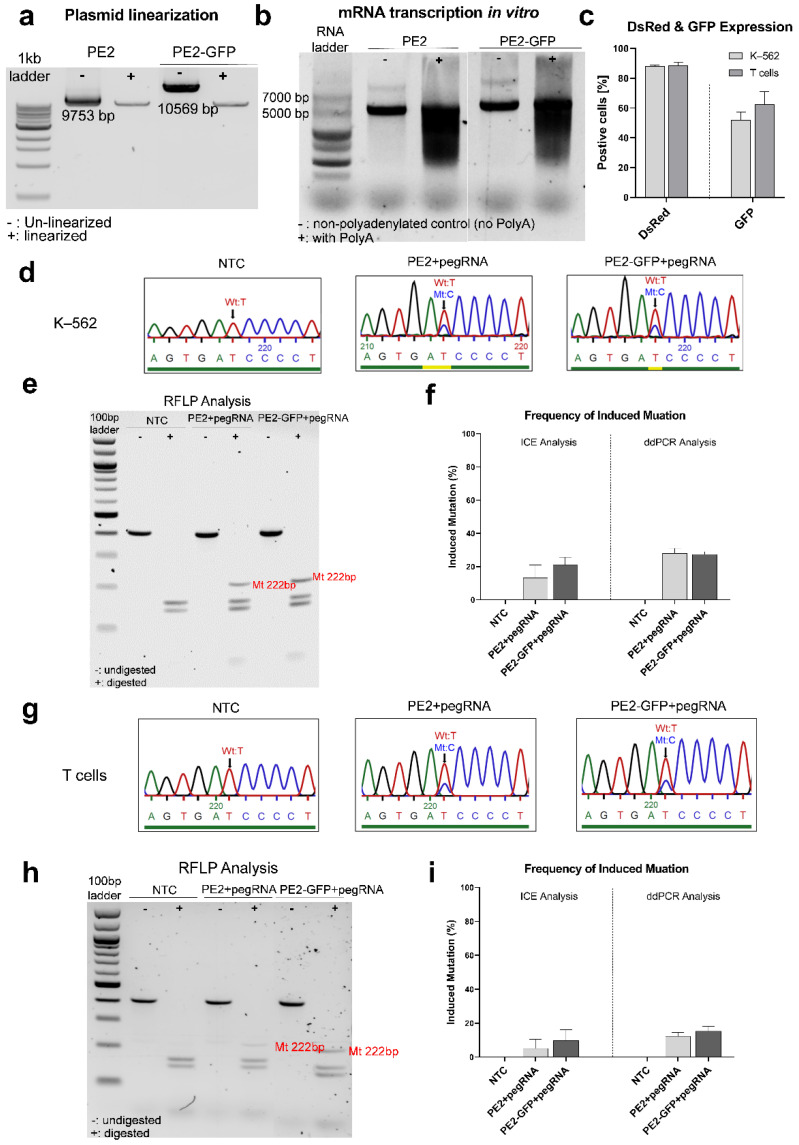
Prime editing in K-562 cells and healthy donors’ T cells to induce the *IL2RG* c.458T>C mutation. Gel visualization of (**a**) linearized plasmids and (**b**) in vitro synthesized mRNAs. Inducing the *IL2RG* c.458T>C mutation in K–562 cells (n = 2) and healthy donors’ T cells (n = 3) was carried out using prime editing with PE2 and PE2-GFP mRNA and pegRNA1. The DsRed and GFP expressions, as transfection controls, were evaluated 24 h after electroporation. (**c**) Up to 88% of the K–562 cells and 91% of the T cells expressed DsRed; 41–62% of the K–562 cells and 47–76% of the T cells expressed GFP. The gDNA was acquired for a genomic analysis of editing efficiency two days post-electroporation. In K–562 cells, (**d**) PE2 mRNA-pegRNA and PE2-GFP mRNA-pegRNA-transfected cells showed the induced mutant base c.458C (the blue, red, green, and black peaks stand in place of bases C, T, A, and G respectively); (**e**) an RFLP analysis showed that a band with the expected size (222 bp) after the digestion of a PCR product (409 bp) by the *DpnII* enzyme for the mutant sequence in the PE-pegRNA-transfected samples. (**f**) The frequency of induced mutation was evaluated using ICE and ddPCR analysis: 26.5 ± 2.5% (PE2 mRNA-pegRNA) and 29.0 ± 1.0% (PE2-GFP mRNA-pegRNA) via ICE analysis; 28.0 ± 3.0% (PE2 mRNA-pegRNA) and 27.5 ± 1.5% (PE2-GFP mRNA-pegRNA) via ddPCR analysis. In the T cells of healthy donors, (**g**) PE2 mRNA-pegRNA and PE2-GFP mRNA-pegRNA transfection samples showed the induced mutant base c.458C; (**h**) an RFLP analysis revealed mutant generation in the edited samples with PE-pegRNA. (**i**) The frequency of induced mutation was 16.7 ± 8.4% (PE2 mRNA-pegRNA) and 21.0 ± 4.0% (PE2-GFP mRNA-pegRNA) via ICE analysis and 13.1 ± 3.0% (PE2 mRNA-pegRNA) and 18.0 ± 5.3% (PE2-GFP mRNA-pegRNA) via ddPCR analysis. Mean ± SEM of biologically independent experiments are provided.

To assess the efficiency of the designed prime editing strategy to edit the point mutation of the *IL2RG* gene, first, we employed this system to induce the *IL2RG* c.458T>C mutation in K–562 cells and healthy donors’ T cells. A pegRNA1 carrying the *IL2RG* c.458T>C mutation was designed. In vitro-transcribed (IVT)-mRNA production was performed after the linearization of PE2 and PE2-GFP plasmids (Figure 5a,b). The PE (PE2/PE2-GFP mRNA)–pegRNA complex was transfected into the cells. Twenty-four hours after transfection, up to 88% of the K–562 cells and 91% of the T cells expressed DsRed mRNA, and GFP expression was positive for 41–62% of K–562 cells and 47–76% of T cells (Figure 5c).

In K–562 cells, the induced mutation (*IL2RG* c.458T>C) was identified using Sanger sequencing (Figure 5d). Moreover, an RFLP analysis revealed a band with 222 bp, which was expected for this mutation in transfected cells (Figure 5e). The quantification by ICE analysis showed a 26.5 ± 2.5% mutation induction in PE2 mRNA-pegRNA-transfected cells and a 29.0 ± 1.0% frequency in PE2-GFP mRNA-pegRNA-transfected cells (Figure 5f). The generated mutation was also supported by ddPCR analysis: the frequency of the induced mutant nucleotide was 28.0 ± 3.0% in PE2 mRNA-pegRNA and 27.5 ± 1.5% in PE2-GFP mRNA-pegRNA-transfected cells (Figure 5f).

In addition, we were able to induce the *IL2RG* c.458T>C mutation in the T cells of healthy donors. The introduced mutant nucleotide was detected using Sanger sequencing (Figure 5g) and RFLP analysis (Figure 5h). The quantification of the mutant nucleotide via ICE analysis presented 16.7 ± 8.4% in PE2 mRNA-pegRNA-transfected cells and 21.0 ± 4.0% in PE2-GFP mRNA-pegRNA-transfected cells. A ddPCR analysis calculated the frequency for induced mutation as 13.1 ± 3.0% in PE2 mRNA-pegRNA-transfected cells and 18.0 ± 5.3% in PE2-GFP mRNA-pegRNA-transfected cells (Figure 5i).

#### 3.3.2. Correcting the Mutant IL2RG in Mosaic T Cells

**Figure 6:**
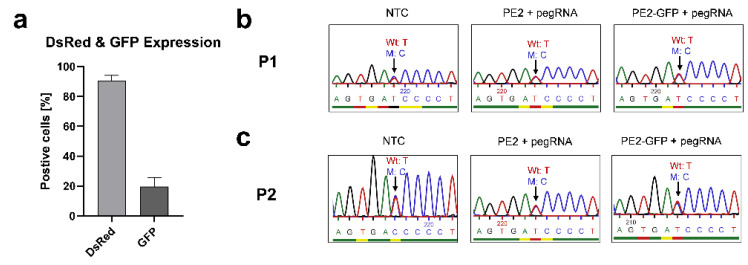
Prime editing in mosaic T cells to correct *IL2RG* c.458T>C mutation. PE (PE2/PE2-GFP mRNA)–pegRNA2 (carrying wild-type base c.458T of *IL2RG*) was transfected to edit the *IL2RG* c.458T>C mutation in the mosaic T cells of the patients (n = 2, P1 and P2). (**a**) Up to 92% and 19% of cells expressed DsRed and GFP, respectively. Sanger sequencing did not exhibit significate differences between the NTC (non-transfected control) and edited samples (transfected with PE2/PE2-GFP mRNA-pegRNA) in either (**b**) P1 and (**c**) P2 (the blue, red, green, and black peaks stand in place of bases C, T, A, and G respectively). Mean ± SEM of biologically independent experiments is shown.

After confirming the functionality of the designed prime editing system, we developed a pegRNA2 carrying the wild-type nucleotide (c.458T) in order to correct the *IL2RG* c.458T>C mutation in the T cells of patients. The T cells were electroporated with a PE (PE2/PE2-GFP mRNA)-pegRNA complex and DsRed mRNA. Up to 92% of cells were positive for DsRed, and 19% of cells expressed GFP (Figure 6a). Nevertheless, Sanger sequencing (Figure 6b,c), ICE analysis, and ddPCR analysis did not detect any further correction in the mosaic T cells of the patients.

## 4. Discussion

In a previous publication, we described the phenotype of three brothers suffering from atypical X-SCID [13]. The novel c.458T>C *IL2RG* mutation, as well as the genetic mosaicism of natural revertant cells with a correction of the mutation, were considered to be one of the main causes for the amelioration of the symptoms. Interestingly, all three brothers exhibited natural reversion in their T cells. The fact that the reversion was not observable in the Sanger sequencing of the PBMCs or, more specifically, in other lymphocyte types (such as NK cells or B cells) and phagocytic cells (like monocytes or macrophages) led us to assume that the natural reversion only occurred in early progenitor T cells and was absent in hematopoietic stem cells. Although the clinical benefit of immune reconstitution in X-SCID patients with reverted mosaicism is improved by the presence of revertant cells [5,6,7,8,9,11], the illness progression of several patients still worsened with age [10,12]. As a result, the stability of revertant cells is not assured. In case these patients lose the support of the revertant mutation in T cells over time, feasible therapeutic strategies should be considered. Thus, we were inclined to investigate the potential therapeutic effects of gene therapy for the treatment of atypical X-SCID patients with somatic reverted mosaicism.

Gene therapy for X-SCID has achieved tremendous advancements in the last two decades. Previous clinical trials of X-SCID gained promising therapeutic benefits using gene therapy-mediated auto-HSCT with retroviral vectors such as gamma-retroviral and lentiviral vectors expressing wild-type γc. However, some of the patients who were treated by this method developed T cell lymphoblastic leukemia (T-ALL) due to the random integration of the vector in the genome, which led to cellular oncogenesis [33,34,35,36]. Recent clinical trials have shown that X-SCID patients treated with gene therapy using second-generation self-inactivating (SIN) vectors have encouraging clinical benefits, including immune reconstitution, without developing leukemia [37,38,39]. Nevertheless, this process requires a longer follow-up time to assess the risk of insertional mutagenesis for SIN vectors [15]. To improve the feasibility and safety of gene therapy in X-SCID patients, scientists have focused on new alternatives, applying gene editing strategies mediated by various nucleases, for instance, zinc finger nucleases (ZFNs) [40,41,42,43], transcription activator-like effector nucleases (TALENs) [44,45], RNA-guided nucleases (CRISPR/Cas) [20,46], and nuclease-free adeno-associated viruses (AAV) [33]. Different studies applying these approaches to correct mutant *IL2RG* have reported positive results. Urnov et al. [42] transfected ZFNs with a donor plasmid targeting exon5 in *IL2RG* and achieved up to 5.3% HDR frequency in CD4^+^ T cells. Lombardo et al. [43] used ZFNs-integrase-defective lentiviral vector (IDLV) to knock in *IL2RG* cDNA containing a GFP expression cassette in Epstein–Barr (EB) virus-transformed B lymphocytes (lymphoblastoids), which obtained 2.4% GFP^+^ cells. Genovese et al. [47] corrected defective *IL2RG* genes in bone marrow CD34^+^ cells from a patient with X-SCID carrying a missense mutation (*IL2RG* c.865C.T; R289X). They showed that 3–11% of the treated cells with ZFN-IDLV-encoding mRNAs expressed GFP. Pavel-Dinu et al. [20] used CRISPR/Cas9-AAV6 to correct mutations in *IL2RG* and achieved 45% targeted integration in the HSPCs of patients with X-SCID. Here, using the CRISPR/Cas9-ssODN approach, we induced the *IL2RG* c.458T>C mutation and the blocking mutation c.459C>A with up to 5% in K–562 cells and 26% in healthy donors’ T cells efficiencies.

In many studies, as well as ours, CRISPR/Cas9-ssODN was selected as one of the strategies to perform gene editing because the CRISPR/Cas9 system has the advantages of versatility and easy usability. Additionally, short single-stranded oligodeoxynucleotide donors (ssODNs) are simple to design and fast and inexpensive to produce [48]. Although the CRISPR/Cas9 system is a highly efficient approach to generate insertions and deletions in the target sequence, the possibility of specific HDR is very low. Most DSB repair is mediated by the NHEJ pathway, resulting in nonspecific insertions and deletions or other mutations [49,50]. As expected, the results of our study also showed a higher indel frequency in comparison with HDR in CRISPR/Cas9-ssODN-edited samples. Additionally, HDR generation depends on the features of ssODNs (such as the length of homology arms, size, orientation, and the blocking mutation), the type of edited cells, and the loci of genes [50,51,52]. Therefore, it is necessary to optimize ssODNs in order to enhance HDR efficiency.

The prime editing strategy was applied in our study as a safer and more efficient approach. Theoretically, prime editing technology can be employed for genome editing in 89% of known genetic variations related to human diseases [22]. Different generations of the prime editing system could further improve editing efficiency in single-nucleotide substitution, small insertion, and deletion [22]. Anzalone et al. [22] applied PE2 to introduce transversion point mutations to different genomic sites and achieved 1.1–28.1% editing efficiency in HEK293T cells. Li et al. [53] introduced point mutations to **α**-synuclein (*SNCA*) gene (A30P) loci in wild-type human pluripotent stem cells (hPSCs) using PE2 in the plasmid, RNP, and mRNA forms and obtained about 5%, 1%, and 26.7% editing efficiency, respectively. These results showed that using a PE2 system in mRNA formatting is more efficient. In this study, we applied the PE2-pegRNA strategy for installing single-nucleotide transversion, which was capable of generating the *IL2RG* c.458T>C mutation in K–562 cells and healthy donor T cells with up to 31% and 26% efficiency, respectively. This would aid in the optimization of the correction approach, as very similar designs would be employed in patient cells but also provided a suitable cellular model for research on the disease. We showed that, in comparison with a CRISPR/Cas9-ssODN system, prime editing is more efficient in generating the *IL2RG* c.458T>C mutation. The result of Sanger sequencing revealed no on-target indel generation in samples edited with prime editing, which is consistent with previous studies [22], suggesting that the induction of indel is a rare event in the prime editing process.

However, applying similar strategies with highly homologous designs to correct the *IL2RG* c.458T>C mutation in the mosaic T cells of patients did not increase the frequency of the wild-type nucleotide. We hypothesize that a revertant mutation in patient T cells hindered a successful outcome for the following reasons:

- The presence of a revertant mutation reduces the editing efficiency. Because the wild-type and mutant sequences with only one nucleotide variation coexist in the targeted population, this hampers specific targeting in both CRISPR and prime editing systems. Moreover, it leads to more difficulty in detecting the corrected nucleotide when the frequency of gene editing is low. Highly sensitive genotyping techniques such as next-generation sequencing would be required to precisely determine the correction efficiency [54]. Nevertheless, we hypothesize that the correction events for the patients’ treated T cells, if any, would be infrequent at best.
- The probability of targeting mutant cells is lower in a mosaic population. Previously, we investigated the levels of γc in patient T cells, finding them to be similar to healthy donors [13]. The similar expression of γc between wild-type/mutant subpopulations and only one amino acid difference in both proteins make fluorescence-activated cell sorter (FACS) inviable; therefore, working with the mosaic population was required. Moreover, the proliferation of patients’ CD3^+^ T cells was impaired compared with healthy donors [13]. According to the genetic analysis, with the in vitro culturing of CD3^+^ T cells from patients, the frequency of the mutant base reduced over time (Figure 1), which is consistent with the finding in previous studies that cells containing revertant mutation have a proliferative advantage [8,55]. These observations demonstrate that mutant cells are hardly capable of proliferating in vitro. This technical limitation implies a lower opportunity to target mutant cells in the heterogeneous population. Higher volumes of blood should have been processed to achieve sufficient cellular material and meet the need of in vitro expansion. In this way, the direct genetic manipulation of T cells could have been pursued, but it was not further investigated for ethical reasons. Similarly, hematopoietic stem cells (HSCs) would have been the most suitable candidates for gene modification to achieve durable immune reconstitution, but patient HSCs were not collected nor used in the present study due to medical ethics.

Based on the mutagenesis results, we hypothesize that our strategies of CRISPR/Cas9-ssODN and prime editing could be feasibly employed in other X-SCID patients with *IL2RG* mutations at the same loci without reversion. However, further technological improvements need to be developed to correct the mutations in somatic reverted mosaicism. The PE3 prime editing approach was not considered for this study since the additional nicking sgRNA would also target the revertant wild-type sequence present in the mosaic population. We hypothesize a more successful outcome could be achieved with a novel PE4 approach (PE2 enzyme, MLH1dn, and pegRNA), as Chen et al. [56] reported a substantial improvement in gene correction efficiency compared with the PE2 strategy in primary T cells, but limited access to patient material makes further optimization experiments inviable.

Importantly, the identification of mosaicisms should be considered in the clinical diagnosis and treatment of genetic disorders. The accurate detection of mosaicism depends on the type of acquired cells (for example, in our case, only T cell subsets presented the revertant mutation) and the sensitivity of the genotyping techniques [57,58]. The diagnosis and identification of genetic diseases are more challenging in mosaicisms due to revertant mutations [58]. For patients with atypical X-SCID, the genomic analysis of multiple immune cell subtypes is needed to determine the presence of reversion. Moreover, state-of-the-art techniques such as next-generation sequencing (NGS), quantitative PCR (q-PCR), and ddPCR should be routinely employed to detect and quantify mosaicism, as they provide more sensitive results [54]. Additionally, exon sequencing and whole-genome sequencing are also commonly used to identify the pathogenic variations of DNA [54]. Hence, patients with atypical clinical symptoms of genopathy should be evaluated by various detection techniques. After a comprehensive evaluation of the detected results, a precise diagnosis and appropriate treatment should be implemented.

## 5. Conclusions

In summary, we induced the specific *IL2RG* mutation in K–562 cells and T cells using the designed CRISPR/Cas9 and prime editing systems. Applying these two strategies to the disease model of X-SCID can improve the precise evaluation of the possible progressions and potential treatments of the disease. According to our results, it is challenging to correct the mutant cells in the presence of mosaicism. We hypothesize our strategies could be more effectively applied to correct *IL2RG* c.458T>C in patient cells without revertant mutations, as well as other mutations in the same locus. Similar strategies involving prime editing and CRISPR/Cas9 systems can provide valuable alternatives for the treatment of X-SCID and inherited immunodeficiencies.

## Data Availability

The data presented in this study are available from the corresponding authors upon reasonable request.

## Supplementary Materials

The following supporting information can be downloaded at https://www.mdpi.com/article/10.3390/genes13122348/s1: Table S1: sgRNA sequence; Table S2: ssODN sequence; Table S3: pegRNA sequence; Table S4: Primer sequence; Table S5: Sequence of primers and probes for ddPCR assay.

## References

[1] Puck J.M., Deschênes S.M., Porter J.C., Dutra A.S., Brown C.J., Willard H.F., Henthorn P.S. (1993). The interleukin-2 receptor γ chain maps to Xq13.1 and is mutated in X-linked severe combined immunodeficiency, SCIDX1. Hum. Mol. Genet..

[2] Gaspar H.B., Qasim W., Davies E.G., Rao K., Amrolia P.J., Veys P. (2013). How I treat severe combined immunodeficiency. Blood.

[3] Kumrah R., Vignesh P., Patra P., Singh A., Anjani G., Saini P., Sharma M., Kaur A., Rawat A. (2020). Genetics of severe combined immunodeficiency. Genes Dis..

[4] Lim C.K., Abolhassani H., Appelberg S.K., Sundin M., Hammarström L. (2019). hypomorphic mutation: Identification of a novel pathogenic mutation in exon 8 and a review of the literature. Allergy Asthma Clin. Immunol. Off. J. Can. Soc. Allergy Clin. Immunol..

[5] Stephan V., Wahn V., Le Deist F., Dirksen U., Broker B., Müller-Fleckenstein I., Horneff G., Schroten H., Fischer A., de Saint Basile G. (1996). Atypical X-linked severe combined immunodeficiency due to possible spontaneous reversion of the genetic defect in T cells. N. Engl. J. Med..

[6] Kawai T., Saito M., Nishikomori R., Yasumi T., Izawa K., Murakami T., Okamoto S., Mori Y., Nakagawa N., Imai K. (2012). Multiple reversions of an IL2RG mutation restore T cell function in an X-linked severe combined immunodeficiency patient. J. Clin. Immunol..

[7] Hsu A.P., Pittaluga S., Martinez B., Rump A.P., Raffeld M., Uzel G., Puck J.M., Freeman A.F., Holland S.M. (2015). IL2RG reversion event in a common lymphoid progenitor leads to delayed diagnosis and milder phenotype. J. Clin. Immunol..

[8] Kuijpers T.W., van Leeuwen E.M.M., Barendregt B.H., Klarenbeek P., aan de Kerk D.J., Baars P.A., Jansen M.H., de Vries N., van Lier R.A.W., van der Burg M. (2013). A reversion of an IL2RG mutation in combined immunodeficiency providing competitive advantage to the majority of CD8+ T cells. Haematologica.

[9] Kury P., Führer M., Fuchs S., Lorenz M.R., Giorgetti O.B., Bakhtiar S., Frei A.P., Fisch P., Boehm T., Schwarz K. (2020). Long-term robustness of a T-cell system emerging from somatic rescue of a genetic block in T-cell development. EBioMedicine.

[10] Okuno Y., Hoshino A., Muramatsu H., Kawashima N., Wang X., Yoshida K., Wada T., Gunji M., Toma T., Kato T. (2015). Late-Onset Combined Immunodeficiency with a Novel IL2RG Mutation and Probable Revertant Somatic Mosaicism. J. Clin. Immunol..

[11] Speckmann C., Pannicke U., Wiech E., Schwarz K., Fisch P., Friedrich W., Niehues T., Gilmour K., Buiting K., Schlesier M. (2008). Clinical and immunologic consequences of a somatic reversion in a patient with X-linked severe combined immunodeficiency. Blood.

[12] Lin C.H., Kuehn H.S., Thauland T.J., Lee C.M., De Ravin S.S., Malech H.L., Keyes T.J., Jager A., Davis K.L., Garcia-Lloret M.I. (2020). Progressive B Cell Loss in Revertant X-SCID. J. Clin. Immunol..

[13] Hou Y., Gratz H.P., Ureña-Bailén G., Gratz P.G., Schilbach-Stückle K., Renno T., Güngör D., Mader D.A., Malenke E., Antony J.S. (2021). Somatic Reversion of a Novel Mutation Resulting in Atypical X-Linked Combined Immunodeficiency. Genes.

[14] Buckley R.H., Schiff R.I., Schiff S.E., Markert M.L., Williams L.W., Harville T.O., Roberts J.L., Puck J.M. (1997). Human severe combined immunodeficiency: Genetic, phenotypic, and functional diversity in one hundred eight infants. J. Pediatr..

[15] Blanco E., Izotova N., Booth C., Thrasher A.J. (2020). Immune Reconstitution After Gene Therapy Approaches in Patients With X-Linked Severe Combined Immunodeficiency Disease. Front. Immunol..

[16] Thornhill S.I., Schambach A., Howe S.J., Ulaganathan M., Grassman E., Williams D., Schiedlmeier B., Sebire N.J., Gaspar H.B., Kinnon C. (2008). Self-inactivating gammaretroviral vectors for gene therapy of X-linked severe combined immunodeficiency. Mol. Ther. J. Am. Soc. Gene Ther..

[17] Zychlinski D., Schambach A., Modlich U., Maetzig T., Meyer J., Grassman E., Mishra A., Baum C. (2008). Physiological promoters reduce the genotoxic risk of integrating gene vectors. Mol. Ther. J. Am. Soc. Gene Ther..

[18] Pai S.-Y., Thrasher A.J. (2020). Gene therapy for X-linked severe combined immunodeficiency: Historical outcomes and current status. J. Allergy Clin. Immunol..

[19] Booth C., Romano R., Roncarolo M.G., Thrasher A.J. (2019). Gene therapy for primary immunodeficiency. Hum. Mol. Genet..

[20] Pavel-Dinu M., Wiebking V., Dejene B.T., Srifa W., Mantri S., Nicolas C.E., Lee C., Bao G., Kildebeck E.J., Punjya N. (2019). Gene correction for SCID-X1 in long-term hematopoietic stem cells. Nat. Commun..

[21] Rozov S.M., Permyakova N.V., Deineko E.V. (2019). The Problem of the Low Rates of CRISPR/Cas9-Mediated Knock-ins in Plants: Approaches and Solutions. Int. J. Mol. Sci..

[22] Anzalone A.V., Randolph P.B., Davis J.R., Sousa A.A., Koblan L.W., Levy J.M., Chen P.J., Wilson C., Newby G.A., Raguram A. (2019). Search-and-replace genome editing without double-strand breaks or donor DNA. Nature.

[23] Flotte T.R., Gao G. (2019). Prime Editing: A Novel Cas9-Reverse Transcriptase Fusion May Revolutionize Genome Editing. Hum. Gene Ther..

[24] Bousso P., Wahn V., Douagi I., Horneff G., Pannetier C., Le Deist F., Zepp F., Niehues T., Kourilsky P., Fischer A. (2000). Diversity, functionality, and stability of the T cell repertoire derived in vivo from a single human T cell precursor. Proc. Natl. Acad. Sci. USA.

[25] Labun K., Montague T.G., Krause M., Torres Cleuren Y.N., Tjeldnes H., Valen E. (2019). CHOPCHOP v3: Expanding the CRISPR web toolbox beyond genome editing. Nucleic Acids Res..

[26] Osborn M.J., Webber B.R., Knipping F., Lonetree C.-l., Tennis N., DeFeo A.P., McElroy A.N., Starker C.G., Lee C., Merkel S. (2016). Evaluation of TCR Gene Editing Achieved by TALENs, CRISPR/Cas9, and megaTAL Nucleases. Mol. Ther. J. Am. Soc. Gene Ther..

[27] Chow R.D., Chen J.S., Shen J., Chen S. (2021). A web tool for the design of prime-editing guide RNAs. Nat. Biomed. Eng..

[28] Lamsfus-Calle A., Daniel-Moreno A., Antony J.S., Epting T., Heumos L., Baskaran P., Admard J., Casadei N., Latifi N., Siegmund D.M. (2020). Comparative targeting analysis of KLF1, BCL11A, and HBG1/2 in CD34 HSPCs by CRISPR/Cas9 for the induction of fetal hemoglobin. Sci. Rep..

[29] Antony J.S., Daniel-Moreno A., Lamsfus-Calle A., Raju J., Kaftancioglu M., Ureña-Bailén G., Rottenberger J., Hou Y., Santhanakumaran V., Lee J.-H. (2022). A Mutation-Agnostic Hematopoietic Stem Cell Gene Therapy for Metachromatic Leukodystrophy. CRISPR J..

[30] Conant D., Hsiau T., Rossi N., Oki J., Maures T., Waite K., Yang J., Joshi S., Kelso R., Holden K. (2022). Inference of CRISPR Edits from Sanger Trace Data. CRISPR J..

[31] Paquet D., Kwart D., Chen A., Sproul A., Jacob S., Teo S., Olsen K.M., Gregg A., Noggle S., Tessier-Lavigne M. (2016). Efficient introduction of specific homozygous and heterozygous mutations using CRISPR/Cas9. Nature.

[32] Medley J.C., Hebbar S., Sydzyik J.T., Zinovyeva A.Y. (2022). Single nucleotide substitutions effectively block Cas9 and allow for scarless genome editing in Caenorhabditis elegans. Genetics.

[33] Hiramoto T., Li L.B., Funk S.E., Hirata R.K., Russell D.W. (2018). Nuclease-free Adeno-Associated Virus-Mediated Il2rg Gene Editing in X-SCID Mice. Mol. Ther. J. Am. Soc. Gene Ther..

[34] Hacein-Bey-Abina S., Von Kalle C., Schmidt M., McCormack M.P., Wulffraat N., Leboulch P., Lim A., Osborne C.S., Pawliuk R., Morillon E. (2003). LMO2-associated clonal T cell proliferation in two patients after gene therapy for SCID-X1. Science.

[35] Hacein-Bey-Abina S., von Kalle C., Schmidt M., Le Deist F., Wulffraat N., McIntyre E., Radford I., Villeval J.-L., Fraser C.C., Cavazzana-Calvo M. (2003). A serious adverse event after successful gene therapy for X-linked severe combined immunodeficiency. N. Engl. J. Med..

[36] Hacein-Bey-Abina S., Garrigue A., Wang G.P., Soulier J., Lim A., Morillon E., Clappier E., Caccavelli L., Delabesse E., Beldjord K. (2008). Insertional oncogenesis in 4 patients after retrovirus-mediated gene therapy of SCID-X1. J. Clin. Investig..

[37] Mamcarz E., Zhou S., Lockey T., Abdelsamed H., Cross S.J., Kang G., Ma Z., Condori J., Dowdy J., Triplett B. (2019). Lentiviral Gene Therapy Combined with Low-Dose Busulfan in Infants with SCID-X1. N. Engl. J. Med..

[38] Hacein-Bey-Abina S., Pai S.-Y., Gaspar H.B., Armant M., Berry C.C., Blanche S., Bleesing J., Blondeau J., de Boer H., Buckland K.F. (2014). A modified γ-retrovirus vector for X-linked severe combined immunodeficiency. N. Engl. J. Med..

[39] De Ravin S.S., Wu X., Moir S., Anaya-O’Brien S., Kwatemaa N., Littel P., Theobald N., Choi U., Su L., Marquesen M. (2016). Lentiviral hematopoietic stem cell gene therapy for X-linked severe combined immunodeficiency. Sci. Transl. Med..

[40] Urnov F.D., Rebar E.J., Holmes M.C., Zhang H.S., Gregory P.D. (2010). Genome editing with engineered zinc finger nucleases. Nat. Rev. Genet..

[41] Schiroli G., Ferrari S., Conway A., Jacob A., Capo V., Albano L., Plati T., Castiello M.C., Sanvito F., Gennery A.R. (2017). Preclinical modeling highlights the therapeutic potential of hematopoietic stem cell gene editing for correction of SCID-X1. Sci. Transl. Med..

[42] Urnov F.D., Miller J.C., Lee Y.-L., Beausejour C.M., Rock J.M., Augustus S., Jamieson A.C., Porteus M.H., Gregory P.D., Holmes M.C. (2005). Highly efficient endogenous human gene correction using designed zinc-finger nucleases. Nature.

[43] Lombardo A., Genovese P., Beausejour C.M., Colleoni S., Lee Y.-L., Kim K.A., Ando D., Urnov F.D., Galli C., Gregory P.D. (2007). Gene editing in human stem cells using zinc finger nucleases and integrase-defective lentiviral vector delivery. Nat. Biotechnol..

[44] Joung J.K., Sander J.D. (2013). TALENs: A widely applicable technology for targeted genome editing. Nat. Rev. Mol. Cell Biol..

[45] Menon T., Firth A.L., Scripture-Adams D.D., Galic Z., Qualls S.J., Gilmore W.B., Ke E., Singer O., Anderson L.S., Bornzin A.R. (2015). Lymphoid regeneration from gene-corrected SCID-X1 subject-derived iPSCs. Cell Stem Cell.

[46] Sander J.D., Joung J.K. (2014). CRISPR-Cas systems for editing, regulating and targeting genomes. Nat. Biotechnol..

[47] Genovese P., Schiroli G., Escobar G., Tomaso T.D., Firrito C., Calabria A., Moi D., Mazzieri R., Bonini C., Holmes M.C. (2014). Targeted genome editing in human repopulating haematopoietic stem cells. Nature.

[48] Azhagiri M.K.K., Babu P., Venkatesan V., Thangavel S. (2021). Homology-directed gene-editing approaches for hematopoietic stem and progenitor cell gene therapy. Stem Cell Res. Ther..

[49] Hsu P.D., Lander E.S., Zhang F. (2014). Development and applications of CRISPR-Cas9 for genome engineering. Cell.

[50] Cong L., Ran F.A., Cox D., Lin S., Barretto R., Habib N., Hsu P.D., Wu X., Jiang W., Marraffini L.A. (2013). Multiplex genome engineering using CRISPR/Cas systems. Science.

[51] Okamoto S., Amaishi Y., Maki I., Enoki T., Mineno J. (2019). Highly efficient genome editing for single-base substitutions using optimized ssODNs with Cas9-RNPs. Sci. Rep..

[52] Antony J.S., Latifi N., Haque A., Lamsfus-Calle A., Daniel-Moreno A., Graeter S., Baskaran P., Weinmann P., Mezger M., Handgretinger R. (2018). Gene correction of HBB mutations in CD34^+^ hematopoietic stem cells using Cas9 mRNA and ssODN donors. Mol. Cell. Pediatr..

[53] Li H., Busquets O., Verma Y., Syed K.M., Kutnowski N., Pangilinan G.R., Gilbert L.A., Bateup H.S., Rio D.C., Hockemeyer D. (2022). Highly efficient generation of isogenic pluripotent stem cell models using prime editing. eLife.

[54] Tsiatis A.C., Norris-Kirby A., Rich R.G., Hafez M.J., Gocke C.D., Eshleman J.R., Murphy K.M. (2010). Comparison of Sanger sequencing, pyrosequencing, and melting curve analysis for the detection of KRAS mutations: Diagnostic and clinical implications. J. Mol. Diagn. JMD.

[55] Miyazawa H., Wada T. (2021). Reversion Mosaicism in Primary Immunodeficiency Diseases. Front. Immunol..

[56] Chen P.J., Hussmann J.A., Yan J., Knipping F., Ravisankar P., Chen P.F., Chen C., Nelson J.W., Newby G.A., Sahin M. (2021). Enhanced prime editing systems by manipulating cellular determinants of editing outcomes. Cell.

[57] Biesecker L.G., Spinner N.B. (2013). A genomic view of mosaicism and human disease. Nat. Rev. Genet..

[58] Aluri J., Cooper M.A. (2021). Genetic Mosaicism as a Cause of Inborn Errors of Immunity. J. Clin. Immunol..

