# Supplementary material for: Challenges in Gene Therapy for Somatic Reverted Mosaicism in X-Linked Combined Immunodeficiency by CRISPR/Cas9 and Prime Editing
Source: Genes (Basel). 2022 Dec 13;13(12):2348. doi: 10.3390/genes13122348 (PMC9777626; doi:10.3390/genes13122348)
Supplement: Supplementary file 1 [file genes-13-02348-s001.zip › genes-2027246-supplementary.pdf]

Table S1: sgRNA sequence

Gene		Sequence (5′→3′)
IL2RG	sgRNA 1	ACATATCTCCAGTGATCCCCTGG
	sgRNA 2	TTAGGTTCTCTGGAGCCCAGGGG
	sgRNA 3	TAGGTTCTCTGGAGCCCAGGGGG
	sgRNA 4	TCTGGAGCCCAGGGGGTCACTGG
	sgRNA 5	TCTGGAGCCCAGGGGATCACTGG
	sgRNA 6	GGTGCAGTACCGGACTGACTGGG
TRAC		GAGAATCAAAATCGGTGAATAAA

* Underline base in sgRNAs of *IL2RG* correspond to mutation; last three nucleotides marked in red is PAM.

Table S2: ssODN sequence

	Sequence (5′→3′)	Aim	Size
ssODN 1	A*T*C*CTGACTTGTCTAGGCCAGGGGAATGACCACAT<br>ATGCACACATATCTCCAGTGACCCCCTGGGCTCCAGA<br>GAACCTAACACTTCACAAACTGAGTGAATCCCAGCTA<br>GAACTGAACTGGAA*C*A*A	inducing mutation	126 nt
ssODN 2	C*C*A*GGGGAATGACCACATATGCACACATATCTCCA<br>GTGACACCCTGGGCTCCAGAGAACCTAACACTTCACA<br>AACT*G*A*G	inducing mutation	79 nt
ssODN 3	A*G*G*AGGTATTAGGGGCACTACCTTCAGGATCCTGA<br>CTTGTCTAGGCCAGGGGAATGACCACATATGCACACA<br>TATCTCCAGTGATCCCCTGGGCTCCAGAGAACCTAAC<br>ACTTCACAAACTGAG*T*G*A	correcting mutation	127 nt
ssODN 4	A*T*C*CTGACTTGTCTAGGCCAGGGGAATGACCACAT<br>ATGCACACATATCTCCAGTGATCCCCTGGGCTCCAGA<br>GAACCTAACACTTCACAAACTGAGTGAATCCCAGCTA<br>GAACTGAACTGGAA*C*A*A	correcting mutation	126 nt

* The marked nucleotides in green aim to correct the mutation, whilst the red ones aim to induce mutation. All of ssODNs were chemically modified by incorporating of 3′phosphonothioate 2'-O-methyl in three terminal nucleotides at both 5′ and 3′ ends.

Table S3: pegRNA sequence

	Sequence (5′→3′)	Aim	Size
pegRNA 1	U*U*A*GGUUCUCUGGAGCCCAGGUUUUAGAGCUAG<br>AAAUAGCAAGUUAAAAUAAGGCUAGUCCGUUAUC<br>AACUUGAAAAAGUGGCACCGAGUCGGUGCAGUG<br>ACCCCCUGGGCUCCAGAG*A*A*C	inducing mutation	121 nt
pegRNA 2	U*A*G*GUUCUCUGGAGCCCAGGGUUUUAGAGCUAG<br>AAAUAGCAAGUUAAAAUAAGGCUAGUCCGUUAUC<br>AACUUGAAAAAGUGGCACCGAGUCGGUGCUCCA<br>GUGAUCCCCUGGGCUCCAG*A*G*A	correcting mutation	122 nt

* The marked nucleotides in red aim to induce mutation and green one aims to correct mutation. Two pegRNAs were chemically modified by incorporating of 3′phosphonothioate 2'-O-methyl in three terminal nucleotides at both 5′ and 3′ ends.

Table S4: Primer sequence

Gene	Item	Sequence (5′→3′)	Amplicon length	Source
IL2RG	Forward	AGGCCACACAGATGCTAAAACT	409 bp	own design
	Reverse	TGCTACATTCACGTCCCTAGT		
TRAC	Forward	ATCACGAGCAGCTGGTTTCT	636 bp	Osborn et al. [28]
	Reverse	CCCGTGTCATTCTCTGGACT		

Table S5: Sequence of primers and probes for ddPCR assay

Gene		Sequence (5′→3′)
IL2RG	Forward primer	TATTAGGGGCACTACCTTC
	Reverse primer	TTGTGAAGTGTTAGGTTCTC
	Edited Probe	/6FAM CCAGGGGATCACTGGA /3IABkFQ/
	Native Dark Probe	CCAGGGGGTCACTGGA
RPP30	Forward primer	AGATTTGGACCTGCGAGCG
	Reverse primer	GAGCGGCTGTCTCCACAAGT
	Probe	/5HEX TTCTGACCT/ZEN/GAAGGCTCTGCGCG/3IABkFQ/

*Edited probe of *IL2RG* gene targets wild-type sequence while native dark probe binds mutant sequence.
